# Comparative Pharmacokinetics and Bioavailability of Three Ephedrines in Rat after Oral Administration of Unprocessed and Honey-Fried Ephedra Extract by Response Surface Experimental Design

**DOI:** 10.1155/2017/2802193

**Published:** 2017-06-19

**Authors:** Yezhe Cheng, Yu Zhang, Hang Xing, Kun Qian, Longshan Zhao, Xiaohui Chen

**Affiliations:** ^1^School of Traditional Chinese Materia Medica, Shenyang Pharmaceutical University, Shenyang 110016, China; ^2^School of Pharmacy, Shenyang Pharmaceutical University, Shenyang 110016, China

## Abstract

Ephedra have been used as a common traditional Chinese medicine for thousands of years. However, the perspiration effect of the unprocessed ephedra was too strong. Clinical trials have shown that processing methods play a critical role in moderating the perspiration property of ephedra according to the needs. A LC-MS/MS method was developed and validated to compare the pharmacokinetic properties of the three ephedrines after oral administration of unprocessed and honey-fried ephedra extract. The contents of honey, frying temperature, and frying time were set at 20%, 116°C, and 7 min by the Box-Behnken response surface method, respectively. In the pharmacokinetics study, the biosamples were pretreated and extracted by protein precipitation method with acetonitrile and separated on an Agilent TC-C_18_ column (250 mm × 4.6 mm, 5 *μ*m) using a mobile phase consisting of 0.1% formic acid methanol and 5 mM ammonium acetate aqueous solution (5 : 95, v/v). All calibration curves were linear (*r* > 0.9932) with lower limits of quantitation (LLOQs) < 12 ng/mL. The mean recoveries of the three analytes were higher than 75%. The pharmacokinetics study indicated that the reduced absorption of ephedrine hydrochloride (EH) and pseudoephedrine hydrochloride (PEH) in honey-fried ephedra group might be the main reason for the moderation of the diaphoretic property.

## 1. Introduction

Ephedra (Mahuang in Chinese), the dried stems of* Ephedra sinica* Stapf,* Ephedra intermedia* Schrenk et C. A. Mey. and* Ephedra equisetina* Bge. from Ephedraceae [1], has been widely used for thousands of years for treatment of allergies, asthma, pneumonia, chills, edema, colds, and fever [2–9]. However, the perspiration effect of the unprocessed ephedra was too strong and would cause the* qi* consuming, body fluid damaging, and imbalance of* qi*-blood and* yin-yang*, especially for the elderly, children, or weak patients. Clinical trials have shown that processing methods play a critical role in moderating the perspiration property of ephedra according to the needs [10]. The traditional processing methods for ephedra are various [11]; honey-fried is one of the most significant processing methods. Honey was natured, sweet, and nontoxic, benefitting for nourishing* yin* and moistening lungs. After processing, the diaphoresis and antitussive and antiasthmatic effect of honey-fried ephedra was gentler [12, 13]. Therefore, preparing the best effect honey-fried ephedra was beneficial to the rational application of honey-fried ephedra and unprocessed ephedra. Most of the processing methods recorded in the literatures were simple subjective description and lacked experimental data support. Hence, it was difficult to prepare the best effect honey-fried ephedra.

Response surface methodology (RSM) was a prominent mathematical and statistical technique. In recent years, RSM was used to optimize, develop, and improve different products or processes and to evaluate the simultaneous effects of several factors. It was initially described and developed by Box and Wilson (1951) [14–21]. Box-Behnken design (BBD), one of the most popular response surface designs, was advantageous due to its widespread use and versatility [22–24]. This design provided efficient solutions and less experimental runs and thus was more economical approach and was used to assess the mathematical relationship between the multiple independent variables so as to determine and optimize the appropriate processing technology of ephedra [22, 25–27].

Based on the traditional processing methods, this paper mainly optimized and determined the processing technology of honey-fried ephedra and compared the variations of effective components. The effects of different contents of honey, frying temperature, and frying time on relative contents of ephedrine hydrochloride (EH), pseudoephedrine hydrochloride (PEH), and methylephedrine hydrochloride (MEH) in honey-fried ephedra have been investigated by using BBD. Better understanding of the processing technology of honey-fried ephedra was beneficial to clinical use of unprocessed ephedra and honey-fried ephedra.

As far as we know, with oral administration as the main route of administration of traditional Chinese Medicine [28], the absorption of active ingredients is the first link to exert therapeutic effect, which should be given enough attention. Up to now, the research on ephedra processing has often focused on the processing technology and the comparison of pharmacological action. In addition, there were few reports on the pharmacokinetics study of bioactive compounds after oral administration of unprocessed ephedra extract and honey-fried extract. Phytochemical and pharmacological studies revealed that EH, PEH exerted significant diaphoretic, antitussive, and antiasthmatic effects, and MEH exerted primary antitussive and antiasthmatic effects. Moreover, these three ephedrines were the main biologically active ingredients in ephedra [8, 27–34].

According to the previous research and experimental conditions, the purpose of this paper was to optimize and determine the processing technology of honey-fried ephedra and to investigate the pharmacokinetic profiles of EH, PEH, and MEH after oral administration of unprocessed ephedra extract and honey-fried extract. In this paper, we present a validated LC-MS/MS method for simultaneous determination of the three ingredients in rat plasma. Additionally, pharmacokinetic parameters of the three ingredients in unprocessed ephedra extract and honey-fried extract were described.

## 2. Materials and Methods 

### 2.1. Materials and Reagents

Ephedra (*Ephedrae Herba*, Batch number 201611) was purchased from Nei Monggol Chifeng Rongxingtang Drug Co., Ltd. (Shenyang, China) and identified by Associate Professor YanNian Wang, a traditional Chinese medicine (TCM) identifying expert from Department of Traditional Chinese Medicine Processing, Shenyang Pharmaceutical University. The contents of EH, PEH, and MEH were quantitatively determined by HPLC and were 9.5, 8.4, and 1.1 mg/g in ephedra, respectively. EH (Batch number 171241-201607; purity, 99.7%), MEH (Batch number 171247-201601; purity, 99.9%), PEH (Batch number 171237-201608; purity, 100%), and internal standard (matrine; purity > 98.5%) were purchased from the National Institute for the Control of Pharmaceutical and Biological Products (Beijing, China). Methanol and acetonitrile of HPLC grade were supplied by Fisher Scientific (Pittsburgh, PA, USA). Ether and isopropyl alcohol (analytical grade) was purchased from Kermel Chemical Reagent Co. Ltd. (Tianjin, China). Distilled water was obtained from Wahaha Co. Ltd. (Hangzhou, China).

### 2.2. Animals

Twelve Male Sprague-Dawley rats weighted 280–300 g were obtained from the Experimental Animal Center of Shenyang Pharmaceutical University. The rats were bred in an air conditioned animal center at a temperature of 25 ± 2°C and a relative humidity of 50 ± 10%, with a natural light-dark cycle for 7 days, and then fasted overnight with only access to water for 12 h before the experiment. Animal study was conducted with strict adherence to the Guideline of Animal Experimentation of Shenyang Pharmaceutical University, and the experimental protocols were approved by the Animal Ethics Committee of the institution.

### 2.3. Response Surface Experimental Design

The effects of different contents of honey, frying temperature, and frying time on relative contents of EH, PEH, and MEH in honey-fried ephedra were investigated by the single factor method, which was utilized by the Box-Behnken response surface method. On the basis of the single factor experimental results, three major influence factors were confirmed, and then a RSM was conducted to design experimental project. As shown in Table 1. The three factors chosen for this study were designated as A, B, and C and prescribed into three levels, adjusted as +1 (maximum), 0 (central), and −1 (minimum).

#### 2.3.1. HPLC-DAD Conditions

The system consists of an Agilent TC-C_18_ column (4.6 mm × 250 mm, 5 *μ*m) with mobile phase of acetonitrile-0.1% phosphoric acid and 0.1% triethylamine water (4 : 96, v/v) at a flow rate of 0.8 mL/min. The column temperature was set at 30°C and the detection wavelength was set at 210 nm.

#### 2.3.2. Standard Solution

**Figure 1:**
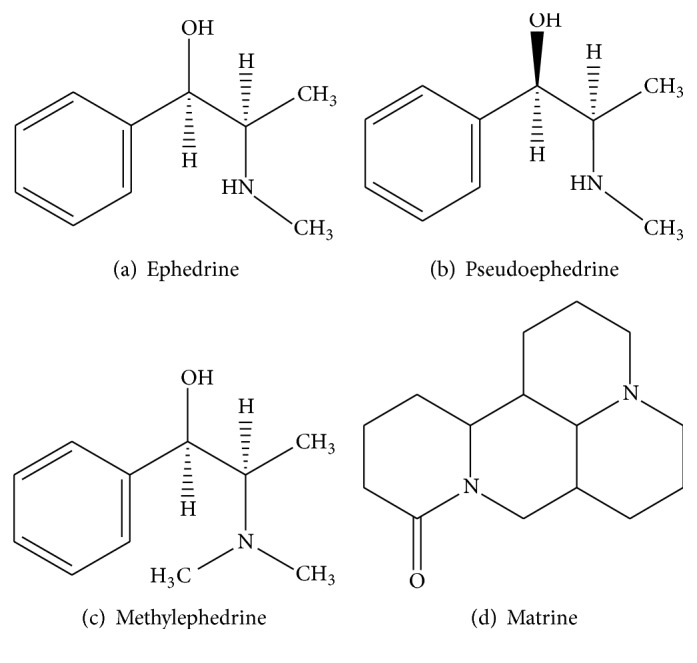
Chemical structures of EH (a), PEH (b), MEH (c), and IS matrine (d).

The stock solutions of EH, PEH, and MEH (1.604 mg/mL, 1.510 mg/mL, and 0.507 mg/mL, resp.) were prepared in methanol. The chemical structures of EH (a), PEH (b), and MEH (c) are shown in Figure 1. A series of mixed working standards having 10–320 ng/mL for EH, 9–302 ng/mL for PEH, and 1–30 ng/mL for MEH were obtained by diluting a mixture of the stock solutions with methanol. All the solutions were stored at −20°C.

#### 2.3.3. Preparation of Samples with Different Processing Conditions

The right amount of samples was mixed well with diluted honey (the contents of honey were 10%, 20%, and 30%) and was moistened for 12 h. Then, the samples were fried with different temperature (110°C, 120°C, and 130°C) and were fried until losing their stickiness (frying time was 6 min, 8 min, and 10 min). Each of the foregoing samples was prepared variously for three until use.

### 2.4. Instruments and LC-MS/MS Conditions

**Figure 2:**
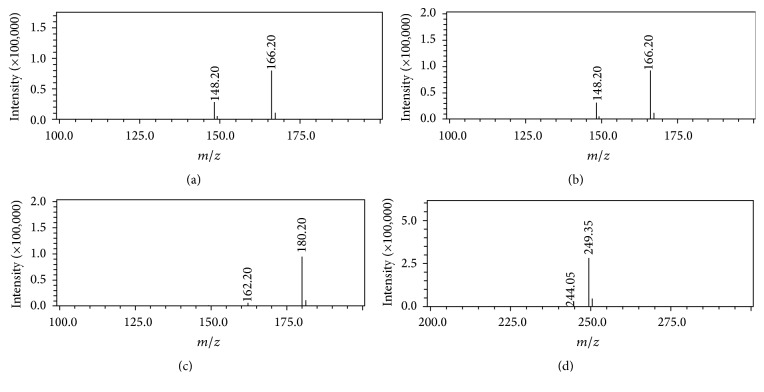
The LC-MS/MS product ion mass spectra: the [M+H]^+^* m*/*z* 166.20 for EH (a), the [M+H]^+^* m*/*z* 166.20 for PEH (b), the [M+H]^+^* m*/*z* 180.20 for MEH (c), and the [M+H]^+^* m*/*z* 249.35 for IS (d).

The liquid chromatographic analysis was performed using a Shimadzu (Japan, Kyoto) LC-MS/MS 2010EV system equipped with electrospray ionization (ESI) interface. The separation was achieved on an Agilent TC-C_18_ column (250 mm × 4.6 mm, 5 *μ*m) at 30°C using methanol (A)-0.1% formic acid and 5 mM ammonium acetate water (B) (5 : 95, v/v) as initial proportion of the isocratic elution. The flow rate was set at 0.8 mL/min and the injection volume was 20 *μ*L. The IS were ionized by electrospray ionization source in positive ion mode under the following source conditions: nebulizing gas 1.5 L/min, desolvation line (DL) temperature 250°C, heat block temperature 200°C, detector voltage 1.75 kV, and other parameters were fixed as the tuning file. Analysis was carried out by SIM mode for EH [M+H]^+^* m*/*z* 166.20, PEH [M+H]^+^* m*/*z* 166.20, MEH [M+H]^+^* m*/*z* 180.20, and IS [M+H]^+^* m*/*z* 249.35. The chemical structures of EH (a), PEH (b), MEH (c), and the IS matrine (d) are shown in Figure 1 and the full-scan mass spectra of four analytes after injection in mobile phase are shown in Figure 2.

### 2.5. Preparation of Ephedra Extract

The powder of unprocessed ephedra (5 g) was extracted twice by refluxing with water (1 : 10, w/v), 1 h for each time. Then the extraction solutions were combined, filtered, and evaporated to dryness. The solution contained the equivalent of approximately 3.0 g of unprocessed ephedra per mL and was stored at −20°C until use. This preparation was the unprocessed ephedra extract. The same procedure was followed for the preparation of the honey-fried ephedra extracts: using the powder of honey-fried ephedra (5 g) to produce honey-fried ephedra extract.

### 2.6. Standard Solution and Quality-Control Samples

The stock solutions of EH, PEH, MEH, and IS were prepared with methanol at concentrations of 1.604 mg/mL, 1.510 mg/mL, 0.507 mg/mL, and 9 *μ*g/mL, respectively. A series of mixed working standards having 40–16,000 ng/mL for EH, 60–15,000 ng/mL for PEH and 30–1,500 ng/mL for MEH were obtained by diluting a mixture of the stock solutions with methanol. In addition, the stock solution of the IS was diluted to a concentration of 900 ng/mL with methanol as working solution. All the solutions were stored at −20°C.

The calibration standards of EH (8, 20, 80, 320, 1280, and 3200 ng/mL), PEH (12, 60, 120, 480, 1200, and 3000 ng/mL), and MEH (6, 15, 30, 75, 150, and 300 ng/mL) were prepared by adding 20 *μ*L of the mixed working standard solution to blank plasma. Three levels of quality-control (QC) samples at concentrations of 20, 256, and 2560 ng/mL for EH, 30, 240, and 2400 ng/mL for PEH and 15, 60, and 240 ng/mL for MEH in plasma were prepared separately in the same method. All of the solutions were stored at −20°C for further study.

### 2.7. Sample Preparation

Prior to analysis, all frozen subject samples including rat plasma samples, calibration standards, and QC samples were thawed and allowed to equilibrate at room temperature. The 100 *μ*L plasma sample spiked with 20 *μ*L IS (matrine 900 ng/mL) and 50 *μ*L sodium carbonate solution (0.1 mol/L) were vortex-mixed for 30 s. The resulting sample was subjected to protein precipitation with 200 *μ*L acetonitrile and then vortex-mixed for 5 min. After centrifugation at 4500 rpm for 10 min, the supernatant was transferred into another new tube and evaporated to dryness at ambient temperature with a gentle steam of nitrogen. Finally, the residue was dissolved with 100 *μ*L initial mobile phase and 20 *μ*L was injected for LC-MS/MS analysis.

### 2.8. Method Validation

#### 2.8.1. Specificity, Linearity, and Lower Limit of Quantification

Specificity was evaluated by comparing chromatograms from blank plasma with those obtained from the corresponding plasma spiked with EH (18.6 min), PEH (20.3 min), MEH (22.7 min), and IS (12.3 min) and plasma samples after oral administration of unprocessed ephedra extract. Linearity was assessed by analyzing the calibration curves (8–3200 ng/mL for EH, 12–3000 ng/mL for PEH, and 6–300 ng/mL for MEH) in plasma by plotting the peak area ratio (analyte/IS) versus the normalized standard concentration of the analytes. The lower limit of quantification (LLOQ) was defined as the lowest concentration of the calibration curve with an accuracy and precision within the recommended ±20% from their nominal values.

#### 2.8.2. Precision and Accuracy

The accuracy and precision were evaluated by analyzing QC samples in six replications at low, medium, and high concentrations per day and over a period of three consecutive days. The precision was expressed as the relative standard deviation percentage (RSD%), while the accuracy was expressed as relative error percentage (RE%).

#### 2.8.3. Extraction Recovery and Matrix Effect

The extraction recovery and matrix effect at three QC concentrations were evaluated in sets of six replicates. The recoveries of three analytes and the IS were determined by comparing the peak areas obtained from the extracted samples with the analytes spiked before and after extraction.

The matrix effect was measured by comparing the peak areas of analytes added to postextracted blank with analytes dissolved in matrix component-free reconstitution solvent.

#### 2.8.4. Stability

The stability of the analytes in spiked rat plasma samples was investigated at three QC levels under different storage conditions: three cycles of freezing at −20°C and thawing, at the storage temperature (−20°C) for 14 days, at room temperature (25°C) for 8 h, and stability and in autosampler (4°C) for 12 h.

### 2.9. Method Application

#### 2.9.1. Drug Administration and Sampling

Animals housed on cages were randomly divided into two groups, with six rats in each group. All the rats were fasted for 12 h, with free access to water prior to the experiments. After giving 3 g/kg of unprocessed ephedra extract and honey-fried ephedra extract (calculated by crude drug) to each group by oral gavage using a stomach tube, about 0.3 mL of the blood samples was obtained from the orbital plexus of the eyes at 0, 0.08, 0.17, 0.33, 0.5, 1, 2, 4, 6, 8, 12, and 24 h which were placed in heparinized tubes and immediately centrifuged at 6000 rpm for 5 min. The separated plasma samples were finally stored at −80°C until analysis.

#### 2.9.2. Pharmacokinetic Analysis

All pharmacokinetic parameters were analyzed using the Drug and Statistics (DAS) 2.1 software package supplied by Chinese Pharmacological Society and expressed as means ± standard deviation (SD). The comparison of pharmacokinetic data of EH, PEH, and MEH was determined by SPSS 19.0 (Statistical Package for the Social Science) via independent samples *t*-tests after their natural logarithmic transformation or the Mann–Whitney test [35, 36]. A value of *P* < 0.05 was considered statistically significant for all the tests. The data are presented as mean ± SD.

## 3. Results and Discussion

### 3.1. Results of Response Surface Experiment

The response surface experiment was designed with 15 test points, of which 12 were the factorial points, and the 3 was the zero points. The zero points were the central points of the region, and it was necessary to repeat the experiment for 3 times to estimate the experimental error. Specific BBD and observed responses were shown in Table 1.

### 3.2. The Establishment of Regression Equation Model and Significance Test

Using the data in Table 1 and statistical software Design-Expert V. 8.0.6 to regress and fit, the contents of EH, PEH, and MEH were taken as the responses *Y*_1_, *Y*_2_, and *Y*_3_. Predicted responses *Y*_1_, *Y*_2_, and *Y*_3_ could be expressed by the following second-order polynomial equations:

(1) Y1=8.40−0.25A−0.73B−1.39C−0.14AB+0.091AC−0.67BC−1.20A2−0.94B2−1.65C2,R2=0.9729,Y2=7.29−0.07A−0.67B−1.18C+0.039AB−0.098AC−0.48BC−1.01A2−0.41B2−1.33C2,R2=0.9671,Y3=0.87−0.021A−0.072B−0.14C+2.500E−003AB+0.032AC−0.066BC−0.11A2−0.12B2−0.18C2,R2=0.8956.

The results indicated that the regression equations were significant; the model had a good fit to the experiment and represented the relationship between the responses and factors. The model could be used to analyse and predict the contents of EH, PEH, and MEH under different processing conditions.

### 3.3. The Analysis and Optimization of Response Surface

**Figure 3:**
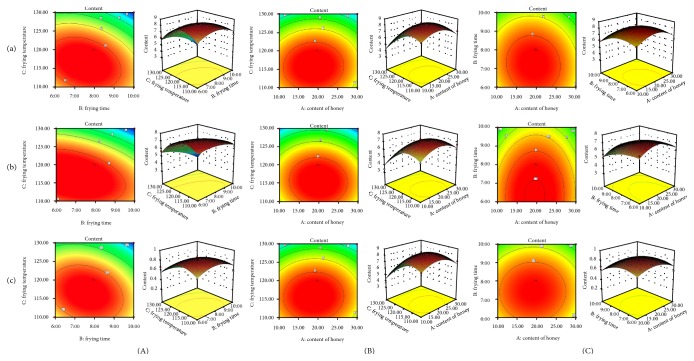
Response surface and contour plot showing interactive effect of different factors on the extraction content of EH (a), PEH (b), and MEH (c). (A) Frying time and frying temperature; (B) content of honey and frying temperature; (C) frying time and content of honey.

According to the regression equation model, the relationship between the responses and the experimental variables can be illustrated graphically to investigate the interactions of the variables and to determine the optimal level of each variable for the maximum response by plotting three-dimensional response surface plots. After investigating the shape of the response surface, the effects of different contents of honey, frying temperature, and frying time on relative contents of EH, PEH, and MEH were analyzed. As shown in Figure 3, the response surface plots directly reflected the influence of each factor and interaction on the response values.

### 3.4. Determination of Optimum Processing Conditions

According to the analysis of statistical software Design-Expert V. 8.0.6, the optimal processing conditions of EH, PEH, and MEH were, respectively, obtained.

The contents of honey, frying temperature, and frying time were set at 18.97%, 116.26°C, and 7.50 min for EH; the contents of honey, frying temperature, and frying time were set at 19.69%, 116.70°C, and 6.76 min for PEH; the contents of honey, frying temperature, and frying time were set at 18.50%, 116.30°C, and 7.60 min for MEH.

Consequently, the optimum processing conditions were established. The contents of honey, frying temperature, and frying time were set at 20%, 116°C, and 7 min, respectively.

All of the honey-fried ephedra mentioned above was prepared with the optimum processing conditions.

### 3.5. Response Surface Experiment Study

One of the strengths of RSM is that it can work well in many cases where there is incomplete knowledge about the study, such as the processing technology of honey-fried ephedra, which lacks theoretical direction and experimental data. The BBD, as a significant response surface design, provides vast quantities of information, avoidance of extreme conditions, and less experiments demand. Therefore, it is easier to process multiple variables and evaluate the interactions between factors [14–18, 22, 25]. Based on the traditional processing methods and limited knowledge of the honey-fried ephedra, we utilized the BBD to develop a mathematical model investigating the relationship between the three parameters (contents of honey, frying temperature, and frying time) in this study. The model, which was considered at multiaspects, has helped to provide reference for the clinical utilization of honey-fried ephedra.

### 3.6. LC-MS/MS Optimization

According to the carboxyl groups of the analytes, the amounts of EH, PEH, MEH, and internal standard (IS) were analyzed in an ESI positive ion mode, and full-scan mass spectra of them after direct injection in the mobile phase were obtained. The results indicated that the ions of four analytes were all [M+H]^+^ ions, and the responses were very stable and showed good linearity in selected ion monitoring (SIM) mode. The quantitative analysis was carried out in SIM as follows: EH [M+H]^+^* m*/*z* 166.20, PEH [M+H]^+^* m*/*z* 166.20, MEH [M+H]^+^* m*/*z* 180.20, and IS [M+H]^+^* m*/*z* 249.35. In order to increase sensitivity, ammonium acetate was tested as a modifier. Addition of 5 mM ammonium acetate enhanced the sensitivity but with the poor peak shape. Thus, different proportions of formic acid (0.05%–0.2%) were added to the mobile phase to improve peak shape. The results indicated that ammonium acetate 5 mM and 0.1% formic acid (v/v) were adopted as the mobile phase for sufficient ionization response, good peak symmetry, and proper retention time for the analytes and IS.

### 3.7. Method Optimization

In order to develop a simple and efficient sample pretreatment to avoid matrix suppression and interference from endogenous plasma components for quantitation of the analytes in rat plasma, a variety of precipitants were investigated. Initially, several conventional liquid-liquid extraction (LLE) procedures were tested by using different extraction solvents such as acetocaustin and ether. Nevertheless, none of them was found suitable to give satisfactory and consistent recovery for all analytes. Therefore, plasma samples were subjected to protein precipitation procedure with acetonitrile and methanol. Furthermore, on account of enhancing the extraction recovery, different concentrations of sodium carbonate were investigated to observe whether they could achieve the purpose or not. Finally, 0.1 mol/L sodium carbonate displayed favorable probably because the analytes were weak alkali; hence, certain alkali added could avoid the hydrolysis of them. Thus, acetonitrile along with 0.1 mol/L sodium carbonate was employed to the pretreatment of the plasma samples.

In selecting the mobile phase procedure, we paid attention to the influence on the chromatographic peak shape and resolution. Acetonitrile was found superior to methanol to get better resolution with some smearing. Because the analytes were weak alkali, the smearing could be alleviated with the poor chromatographic peak shape after using 5 mM ammonium acetate buffer. The addition of 0.1% formic acid and 5 mM ammonium acetate buffer was performed to obtain higher response, better chromatographic peak shape, and shorter run time.

### 3.8. Method Validation

**Figure 4:**
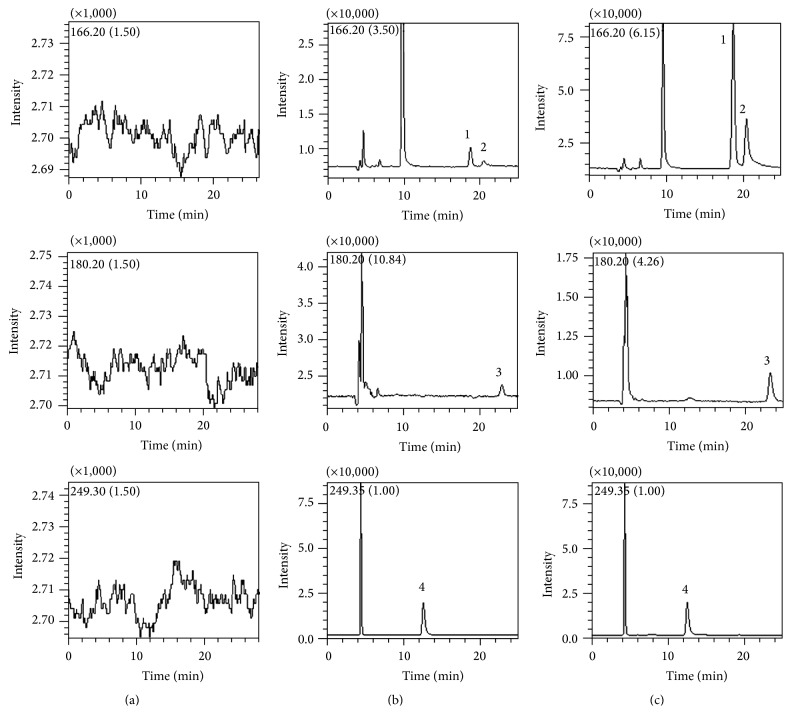
Representative chromatograms of blank rat plasma (a); blank rat plasma spiked with the three analytes at LLOQ and IS (b); rat plasma after 1.5 h after oral administration of the unprocessed ephedra extract (c). Peak 1: EH; 2: PEH; 3: MEH; 4: IS.

Typical chromatograms obtained from blank plasma, blank plasma spiked with analytes (at LLOQ) and IS, and rat plasma samples after 1.5 h oral administration of the unprocessed ephedra extract are shown in Figure 4. No endogenous interference or ion suppression was observed at the retention times of analytes and IS.

The calibration curves exhibited good linearity with the coefficients of correlation (*r*) better than 0.993. The precision and accuracy at the LLOQ for all the analytes were less than 12% and within 11.5, respectively. Intraday and interday precision and accuracy of all the analytes were excellent within acceptance criteria (15%). Linear ranges, slope, intercept, LLOQ, and correlation coefficients obtained from typical calibration curves are shown in Table 2. Intraday and interday precision and accuracy for all the analytes are listed in Table 3.

The recoveries of EH, PEH, MEH, and IS were 77.8–82.0%, 81.9–85.9%, 79.6–88.3%, and 92.8% at different concentration levels, respectively, which proved that the process of extraction was consistent, precise, and reproducible (Table 3). Results of matrix effects (Table 3) indicated that no significant ion suppression or enhancement was observed for the analytes.

The RSD of the stability precisions were less than 15%, and the accuracy ranged from 85% to 115%. The results showed that the three analytes stored within the conditions of the stability tests as mentioned above have been proved to be stable.

### 3.9. Pharmacokinetics Studies

**Figure 5:**
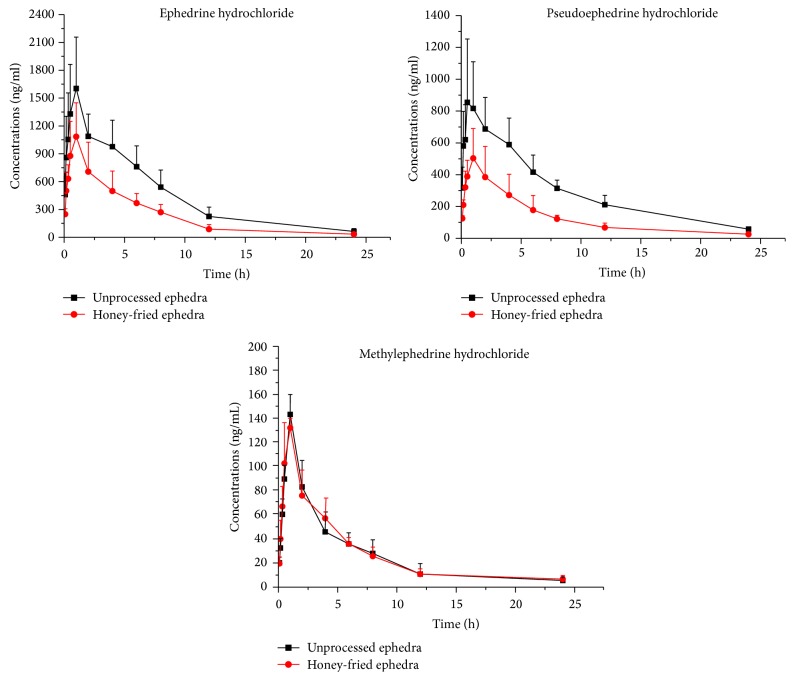
The mean concentration-time curves for EH, PEH, and MEH in rat plasma after oral administration of unprocessed ephedra extract and honey-fried ephedra extract. Each point represents mean ± SD (*n* = 6).

The developed method has been successfully applied to the comparative pharmacokinetics study of EH, PEH, and MEH in rat plasma after oral administration of unprocessed ephedra extract and honey-fried ephedra extract. The concentration-time curves (mean ± SD) of EH, PEH, and MEH are shown in Figure 5 and the corresponding pharmacokinetic parameters are listed in Table 4. As shown in Table 4 and Figure 5, the main differences in pharmacokinetics of EH, PEH, and MEH were *C*_max_, *T*_max_, and AUC. Remarkable decreases (*P* < 0.05) in *C*_max_ and *T*_max_ value of PEH (954.8 ± 307.8 versus 510.5 ± 189.6 *μ*g/L) and (0.7 ± 0.4 versus 1.2 ± 0.4 h) were observed compared with the unprocessed ephedra extract. After oral administration of honey-fried ephedra extract, AUC_0−*t*_ and AUC_0−*∞*_ of the EH and PEH decreased remarkably (*P* < 0.01), as compared with unprocessed ephedra extract. However, there were no significant differences in AUC_0−*t*_, AUC_0−*∞*_, and *C*_max_ values of MEH between the unprocessed ephedra extract and honey-fried ephedra extract. The results indicated that oral administration of honey-fried ephedra extract could lead to poor absorption (AUC) for EH and PEH, when compared with unprocessed ephedra extract. Besides, there was no significant difference in *T*_max_ and *t*_1/2_ of EH and MEH from the ephedra and honey-fried ephedra group (Table 4).

To our knowledge, little research has been reported on the pharmacokinetics of unprocessed ephedra and honey-fried ephedra. In previous reports, other analytical methods, including HPLC and GC-MS, have been applied to evaluate the chemical constitutions before or after processing and the comparison of pharmacological action. Due to limitations of the analysis method or the research directions, the above methods could not fully illustrate the processing mechanism. Therefore, a specific, sensitive, and accurate LC-MS/MS assay for the quantification of EH, PEH, and MEH in rat plasma was developed and validated. The method has been successfully applied to the pharmacokinetic study of EH, PEH, and MEH in the rats after oral administration of unprocessed ephedra extract and honey-fried ephedra extract.

The absorption of EH, PEH exerted significant effect on diaphoresis and general effect on relieving cough and asthma; meanwhile, the absorption of MEH exerted primary effect on relieving cough and asthma. In addition, the pharmacokinetics results (Figure 5) showed that the possible reason for the reduced absorption of EH and PEH in honey-fried ephedra group might be the main reason for the decrease of the bioavailability, which confirmed the theory of that honey-fried ephedra extract could moderate the diaphoretic property of the unprocessed ephedra extract obviously. Meanwhile, there was no significant difference in the absorption of MEH between the unprocessed ephedra extract and honey-fried ephedra extract group. Hence, honey-fried ephedra extract not only eliminates the effect of diaphoresis but also weakens the effect of relieving cough and asthma. The theory that honey-fried ephedra extract could be used for treatment of the elderly, children, or weak patients with cough and asthma exclusively was confirmed.

## 4. Conclusions

RSM was confirmed to be a useful technique for the optimization to describe the honey-fried ephedra processing process and to identify experimental variables (different contents of honey, frying temperature, and frying time). After applying response surface experimental design, we optimized and determined the processing technology of the best effect honey-fried ephedra. Furthermore, the developed LC-MS/MS method was found to meet the requirements of pharmacokinetic studies of the EH, PEH, and MEH in rat plasma after oral administration of unprocessed ephedra extract and honey-fried ephedra extract. Results of this study demonstrated that pharmacokinetic behaviors of EH, PEH, and PEH were different after honey-fried ephedra and unprocessed ephedra were administered. These results may be helpful for the application of ephedra and honey-fried ephedra in clinical therapy.

## Supplementary Material

The information on biosynthesis of ephedrine or pseudoephedrine by ephedra.

## Figures and Tables

**Table 1:** Box-Behnken design and observed response.

Number	A	B	C	*Y* content (mg/g)		
	Content of honey (%)	Frying time (min)	Frying temperature (°C)	EH *Y*_1_	PEH *Y*_2_	MEH *Y*_3_
1	10	10	120	6.291	5.551	0.673
2	20	6	130	6.243	5.722	0.669
3	20	8	120	8.419	7.279	0.866
4	20	6	110	7.103	6.753	0.713
5	20	10	130	3.182	3.398	0.284
6	20	10	110	6.733	6.362	0.590
7	30	8	130	3.958	3.732	0.424
8	20	8	120	8.394	7.307	0.861
9	20	8	120	8.403	7.315	0.872
10	30	10	120	5.019	4.883	0.560
11	20	8	120	8.376	7.286	0.870
12	10	8	130	3.800	3.462	0.328
13	10	8	110	7.310	5.985	0.787
14	30	8	110	7.146	6.646	0.756
15	30	6	120	6.516	6.113	0.590
16	20	8	120	8.419	7.286	0.870
17	10	6	120	7.237	6.937	0.713

**Table 2:** Linear regression data of all the analytes in rat plasma.

	Linear range (ng/mL)	Slope	Intercept	*r*	LLOQ (ng/mL)		
						RE%	RSD%
EH	8–3200	8.2 × 10^−3^	1.7 × 10^−3^	0.9952	8	6.3	7.2
PEH	12–3000	4.7 × 10^−4^	5.0 × 10^−4^	0.9932	12	−3.5	5.8
MEH	6–300	4.4 × 10^−4^	2.0 × 10^−3^	0.9935	6	4.9	8.3

**Table 3:** Precision, accuracy, recovery, and matrix effect for analyses of EH, PEH, and MEH in rat plasma.

	Concentration (ng/mL)	Intraday RSD (%)	Interday RSD (%)	Accuracy RE (%)	Recovery (%)	Matrix effect (%)
EH	20	4.7	8.4	2.9	77.8 ± 4.6	89.3 ± 2.9
	256	3.0	3.4	−5.6	82.0 ± 4.8	96.6 ± 1.7
	2560	6.7	10.9	−4.6	79.3 ± 1.4	104.0 ± 2.0

PEH	30	6.1	11.5	−4.1	83.4 ± 5.0	87.8 ± 4.1
	240	5.2	9.3	2.0	85.9 ± 3.7	102.1 ± 4.9
	2400	7.3	8.4	−4.4	81.9 ± 2.4	90.7 ± 3.1

MEH	15	9.6	6.8	9.6	79.6 ± 4.7	97.6 ± 4.8
	60	6.7	10.4	−4.1	85.3 ± 3.8	88.4 ± 5.1
	240	9.8	7.4	6.7	88.3 ± 3.1	94.0 ± 5.3

**Table 4:** The rat plasma concentrations of EH, PEH, and MEH after oral administration of unprocessed ephedra extract and honey-fried ephedra extract (mean ± SD, *n* = 6).

Data	Unit	EH		PEH		MEH	
		Unprocessed ephedra	Honey-fried ephedra	Unprocessed ephedra	Honey-fried ephedra	Unprocessed ephedra	Honey-fried ephedra
AUC_0−*t*_	*μ*g/L h	10862.4 ± 3168.2	5805.9 ± 1741.2^*∗∗*^	7115.1 ± 1618.7	3145.3 ± 952.4	644.6 ± 163.9	655.2 ± 104.4
AUC_0−*∞*_	*μ*g/L h	11206.8 ± 3224.8	5980.8 ± 1778.1^*∗∗*^	7624.8 ± 1702.0	3438.2 ± 1062.4	687.4 ± 213.9	678.7 ± 101.7
*C*_max_	*μ*g/L	1631.2 ± 522.6	1085.0 ± 363.0	954.8 ± 307.8	510.5 ± 189.6^*∗∗*^	142.8 ± 17.1	136.2 ± 11.9
*t*_1/2_	h	4.4 ± 1.2	4.3 ± 1.8	6.3 ± 0.4	6.7 ± 2.9	5.4 ± 2.5	4.7 ± 1.6
*T*_max_	h	0.9 ± 0.2	1.0 ± 0.0	0.7 ± 0.4	1.2 ± 0.4^*∗*^	1.0 ± 0.0	0.9 ± 0.2

^*∗∗*^ *p* < 0.01 and ^*∗*^*p* < 0.05 (compared to unprocessed ephedra extract group rats).
