# Supplementary material for: Comparative Pharmacokinetics and Bioavailability of Three Ephedrines in Rat after Oral Administration of Unprocessed and Honey-Fried Ephedra Extract by Response Surface Experimental Design
Source: Evid Based Complement Alternat Med. 2017 Jun 19;2017:2802193. doi: 10.1155/2017/2802193 (PMC5494778; doi:10.1155/2017/2802193)
Supplement: Supplementary file 1 — The information on biosynthesis of ephedrine or pseudoephedrine by ephedra. [file 2802193.f1.pdf]

Information on biosynthesis of ephedrine or pseudoephedrine by ephedra:

Biosynthesis of ephedrine and pseudoephedrine by ephedra begins with L-phenylalanine (Phe). Phe is the initial precursor of ephedrine and pseudoephedrine and catalyzed by phenylalanine ammonia lyase (PAL) [S1]. Recent studies suggest that there are at least two possible pathways of Phe side chain- shortening in ephedra benzoic acid biosynthesis: β-oxidative CoA-dependent and non-β-oxidative CoA-independent routes [S2, S3]. A proposed biosynthesis pathway for the biosynthesis of ephedrine and pseudoephedrinein in ephedra is described in Figure S1 [S4-S8]. Figure and references for details can be found in supporting information.

In the β-oxidative CoA-dependent pathway, the first step is the formation of cinnamoyl-CoA, which is catalyzed by cinnamate: CoA ligase. Subsequently, a hydratase converts cinnamoyl-CoA to 3-hydroxy-3-phenylpropanoyl-CoA, and a dehydrogenase catalyzes the 3-hydroxy-3-phenylpropanoyl-CoA into 3-oxo-3-phenylpropanoyl-CoA. In the end, 3-ketoacyl-CoA thiolase catalyzes the formation of benzoyl-CoA (Figure S1) [S6-S8].

In the non-β-oxidative CoA-independent routes, cinnamic acid was converted to 3-hydroxy-3-phenylpropionic-aicd under the catalysis of a hydratase. After retro-aldol cleavaging, 3-hydroxy-3-phenylpropionic-aicd was converted to benzaldehyde. Subsequently, two different dehydrogenase (benzaldehyde dehydrogenase and aldehyde oxidases 4) catalyze the formation of benzoic acid from benzaldehyde. After undergoing condensation with pyruvate, benzaldehyde, benzoyl-CoA and/or benzoic acid was catalyzed by a ThDP-dependent carboligase to form 1-phenylpropane-1,2-dione. Transamination of 1-Phenylpropane-1,2-dione yields (S)-cathinone, which is reduced to (1S,2S)-pseudonorephedrine and (1R,2S)-norephedrine. In the end, N-Methylation of these two ephedrines completes the pathway in ephedra (Figure S1) [S4, S5, S7, S8].

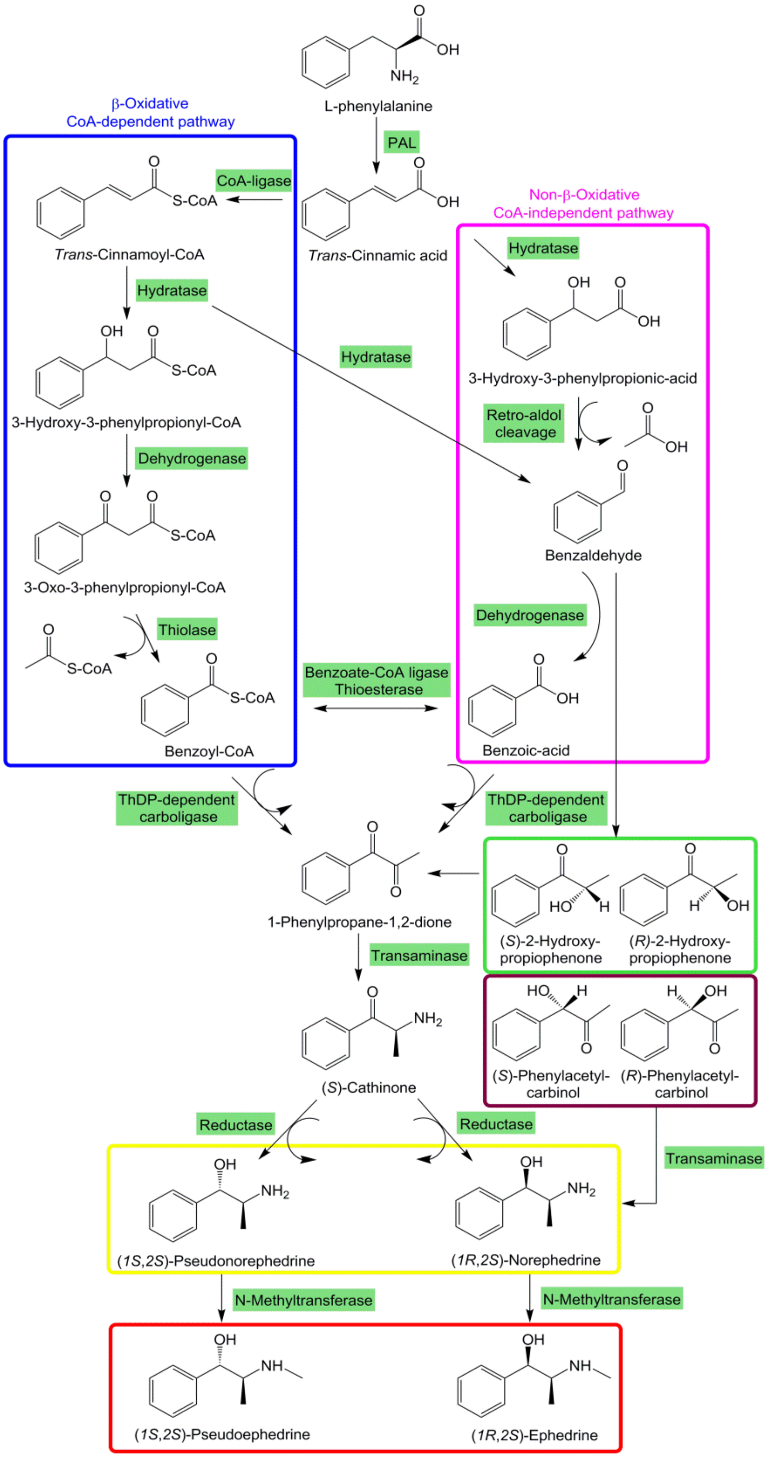

FIGURE S1. Proposed pathways for the biosynthesis of ephedrine and pseudoephedrinein in ephedra. A β-oxidative CoA-dependent pathway of L-phenylalanine side chain-shortening is shown in blue boxes, however a non-β-oxidative CoA-independent pathway is shown in pink boxes. Abbreviations: PAL, phenylalanine ammonia lyase; CoA, Coenzyme A; ThDP, thiamine diphosphate.

References

[S1]. T. Okada, M. Mikage, and S. Sekita. "Molecular characterization of the phenylalanine ammonia-lyase from Ephedra sinica," *Biological & Pharmaceutical Bulletin, vol.* 31, no. 12, pp. 2194-2199.

[S2]. J. Boatright, F. Negre, X. Chen, et al. "Understanding in Vivo Benzenoid Metabolism in Petunia Petal Tissue," *Plant Physiology, vol.* 135, no. 4, pp. 1993-2011.

[S3]. A.V. Qualley, J.R. Widhalm, F. Adebesin, C.M. Kish, and N. Dudareva. "Completion of the core β-oxidative pathway of benzoic acid biosynthesis in plants," Proceedings of the National Academy of Science, vol. 109, no. 40, p. 16383.

[S4]. P.J. Facchini. "ALKALOID BIOSYNTHESIS IN PLANTS: Biochemistry, Cell Biology, Molecular Regulation, and Metabolic Engineering Applications," Annual Review of Plant Biology, vol. 52, no. 52, p. 29.

[S5]. M.C.L. †, D.A.N. ‡, Y.K. †, et al. "Involvement of snapdragon benzaldehyde dehydrogenase in benzoic acid biosynthesis," Plant Journal for Cell & Molecular Biology, vol. 59, no. 2, pp. 256–265.

[S6]. L.V.D. Fits and J. Memelink. "ORCA3, a jasmonate-responsive transcriptional regulator of plant primary and secondary metabolism," Science, vol. 289, no. 5477, pp. 295-297.

[S7]. T. Unver, R.A. Groves, J.M. Hagel, et al. "Transcriptome Profiling of Khat (Catha edulis) and Ephedra sinica Reveals Gene Candidates Potentially Involved in Amphetamine-Type Alkaloid Biosynthesis," Plos One, vol. 10, no. 3, p. e0119701.

[S8]. G.H. Zhang, N.H. Jiang, W.L. Song, C.H. Ma, S.C. Yang, and J.W. Chen. "De novo Sequencing and Transcriptome Analysis of Pinellia ternata Identify the Candidate Genes Involved in the Biosynthesis of Benzoic Acid and Ephedrine," Front Plant Sci, vol. 7, p. 1209.
